# Typhoid after Vaccination

**Published:** 1915-01

**Authors:** 


**Typhoid After Vaccination.** J. G. Gaither, J. A. M. A., Oct. 10, 1914, reports five cases, developing 9-23 months after anti-typhoid inoculation. Three were severe. This series occurred among about 1000 vaccinated. The Secretary of the Kentucky State Board of Health claims that 6739 vaccinations were made without subsequent typhoid. We have previously called attention to a fallacy of the statistic argument with regard to typhoid or almost any other inoculation: namely that the persons volunteering for such protection are most likely to practice general prophylactic measures. The great value of anti-typhoid vaccination has been established in spite of any allowance along such lines of argument but it is not claimed that the method is either permanent or infallible. It is of the utmost importance to have prompt reporting and rigid investigation of all cases of typhoid after vaccination in order to know just what proportion of failures is inevitable and to establish a time limit with regard to re-vaccination.

